# A qualitative study among patients with an inherited retinal disease on the meaning of genomic unsolicited findings

**DOI:** 10.1038/s41598-021-95258-2

**Published:** 2021-08-04

**Authors:** Marlies Saelaert, Heidi Mertes, Tania Moerenhout, Caroline Van Cauwenbergh, Bart P. Leroy, Ignaas Devisch, Elfride De Baere

**Affiliations:** 1 Department of Public Health and Primary Care, Philosophy of Medicine and Ethics Research Group, Ghent University, Campus Heymans (UZ Gent), Corneel Heymanslaan 10 – Building 6K3, 9000 Ghent, Belgium; 2 Department of Philosophy and Moral Sciences, Bioethics Institute Ghent, Ghent University, Ghent, Belgium; 3 Bioethics Centre, University of Otago, Dunedin, New Zealand; 4 Department of Philosophy and Moral Sciences, Ghent University, Ghent, Belgium; 5 Department of Ophthalmology, Ghent University Hospital, Ghent, Belgium; 6 Department of Head and Skin, Ghent University, Ghent, Belgium; 7 Center for Medical Genetics Ghent (CMGG), Ghent University Hospital, Ghent, Belgium; 8 Division of Ophthalmology & Center for Cellular & Molecular Therapeutics, The Children’s Hospital of Philadelphia, Philadelphia, PA USA; 9 Department of Biomolecular Medicine, Ghent University, Ghent, Belgium

**Keywords:** Clinical genetics, Medical ethics

## Abstract

Exome-based testing for genetic diseases can reveal unsolicited findings (UFs), i.e. predispositions for diseases that exceed the diagnostic question. Knowledge of patients’ interpretation of possible UFs and of motives for (not) wanting to know UFs is still limited. This lacking knowledge may impede effective counselling that meets patients’ needs. Therefore, this article examines the meaning of UFs from a patient perspective. A qualitative study was conducted and an interpretative phenomenological analysis was made of 14 interviews with patients with an inherited retinal disease. Patients assign a complex meaning to UFs, including three main components. The first component focuses on result-specific qualities, i.e. the characteristics of an UF (inclusive of actionability, penetrance, severity and age of onset) and the consequences of disclosure; the second component applies to a patient’s lived illness experiences and to the way these contrast with reflections on presymptomatic UFs; the third component addresses a patient’s family embedding and its effect on concerns about disease prognosis and genetic information’s family relevance. The complex meaning structure of UFs suggests the need for counselling procedures that transcend a strictly clinical approach. Counselling should be personalised and consider patients’ lived illness experiences and family context.

## Introduction

Genetic testing by exome sequencing (ES) is increasingly implemented as an efficient technique to diagnose monogenic diseases^1, 2^. ES is able to simultaneously sequence a vast number of genetic regions and, hence, particularly appropriate for identifying the cause of genetically heterogeneous conditions that may be caused by pathogenic variants in multiple genes^3–5^. Since ES may virtually analyse all protein-coding genes, molecular findings may be identified beyond the test’s diagnostic aim. These diagnostically unrelated findings can be unintentionally discovered as unsolicited findings (UFs) or actively pursued as secondary findings (SFs)^6, 7^. Patients and lay people have shown a strong interest in UFs and SFs and they generally would prefer the disclosure of many types of results, including findings associated with an increased cancer risk, early-onset conditions or a carrier status of recessive conditions^3, 8–10^. Some studies have indicated a predominant interest in medically actionable findings^11–14^, meaning results for which a medical treatment or prevention is available that could improve the outcome of the associated condition^15^. However, many people would also want to receive medically non-actionable UFs and SFs, associated with, for instance, progressive neurodegenerative conditions or multifactorial conditions^3, 8, 16, 17^. Many people interpret actionability in a broad sense and refer to lifestyle changes, the psychological value of genetic and genomic information and the value of knowing in itself^11, 12, 17–19^. Nevertheless, not everyone wants to receive diagnostically unrelated findings. Reasons for not wanting to know are the potential costs of additional testing, the complexity of results, a strict focus on diagnostic results or a distrust in the healthcare system^12^. The most common reason for not wanting to receive UFs and SFs is the risk of psychological harm and distress^3, 13, 16, 18^. This psychological risk has specifically been associated with results without clear pathologic significance and with medically non-actionable findings^11, 12, 20^. Nevertheless, lay people generally suppose that refusing the disclosure of UFs and SFs may be more harmful than receiving these results^10, 16, 17, 19^.

Despite current research on patient preferences regarding UFs and SFs, many questions remain unanswered. Firstly, research has often focused on the perspective of cancer patients^3, 11, 14, 20^. This is undeniably an important group of stakeholders who are potentially confronted with UFs and SFs. Patients with different conditions and illness experiences may, however, have different interests regarding genomic results^18, 19^. Secondly, patients have frequently been asked to indicate the categories of UFs and SFs they would like to receive but it is still unclear how they exactly interpret these categories^3, 9, 19^. Most attention has been paid to patients’ perspectives on (medical) actionability but also a condition’s other characteristics should be explored. Thirdly, little is known about the underlying motives for patients’ preferences regarding the disclosure of UFs and SFs. The opportunity for (future) medical decision-making and lifestyle adjustments, as well as the avoidance of psychological distress are important motives but the possibility of other reasons should be explored. Patients’ illness experiences and their family history of disease may affect their perspectives on UFs and SFs but these suggestions require further investigation^10, 12, 19–21^. Limited knowledge of patients’ perspectives and interpretations concerning UFs and SFs may hinder effective counselling sessions that actually meet patients’ needs. In response to these concerns, this article will profoundly examine patients’ interpretation of UFs and SFs, as well as underlying motives for these interpretations and associated preferences (not) to know. Specifically, the meaning of UFs will be investigated among patients with an inherited retinal disease (IRD). This in-depth analysis may contribute to a more effective counselling and more tailored care for patients who are diagnostically tested for various genetic conditions.

## Methods

### Design

The design and analysis of this qualitative study are based on the method of interpretative phenomenological analysis (IPA)^22^. IPA aims to clarify personal meanings of lived experiences or specific objects (‘phenomena’) in a homogeneous group of people^22^. This method is frequently used to understand subjective experiences in healthcare and health psychology and it has also been applied in patients' interpretation of genetic results^23, 24^.

### Recruitment and data collection

Participants were recruited by purposive sampling. People could be included in the study if they had received a diagnosis of an IRD, had been genetically tested, were at least 18 years old and were able to fluently speak Dutch. IRDs represent a large group of clinically and genetically heterogeneous eye disorders^25^. To date, IRDs have been associated with pathogenic variants in over 270 disease genes, making ES-based testing a suitable approach for genetic testing^26^. With the progress of promising gene-based therapies for IRD, a definite genetic diagnosis in IRD is particularly important^27, 28^. Based on the inclusion criteria, E.D.B., B.P.L. and C.V.C. (a geneticist, ophthalmic geneticist and molecular geneticist) selected eligible participants. Eligible patients were contacted by B.P.L. and E.D.B. and were informed about the study. Sixteen patients who were potentially interested to participate, were contacted by telephone by M.S.; they were given additional information and asked for participation. Eventually, fourteen patients (ten women and four men), aged between 23 and 51, agreed to participate and were interviewed. Thirteen of them had received positive genetic test results.

M.S. conducted all in-depth interviews. Interviews took place at the participant’s house or in a room at the university (hospital), depending on participants’ preferences. All interviews were conducted between January 2017 and February 2018 and lasted between 50 and 150 min.

A semi-structured interview guide was used for all interviews. At the time of the interviews, when people were diagnostically tested in Belgian centres for medical genetics, it was not possible to ask for actively pursued SFs. For this reason, interviews were mainly focused on UFs. At the start of the interview, UFs were briefly explained and described as additional genetic and health-related results that are unrelated to IRD. A practice of SFs was also addressed during the interviews but generally, the focus was more on the diagnostically unrelated character of additional findings than on their accidental or active discovery.

Examples of open-ended questions asked during the interview are “How does your experience of IRD affect your daily life?”, “How did you experience the genetic testing process?”, “How do you consider the value or importance of genetic information?”, “How do/did your experiences with IRD and genetic testing affect your family relations, social life, and, if applicable, decisions on children?”, “What are your spontaneous thoughts about additional genetic test results that are not related to IRD?”, “Which kind of results would you be interested in?”, and “How would you expect these results to affect your personal life or family relations?”. To specify questions on UFs and support participants’ reflections, sensitising concepts regarding characteristics of UF-associated conditions were presented. These concepts included (medical) actionability, penetrance (i.e. the probability that a variant will express the associated condition), estimated age of onset, impact on reproduction, and severity, among others. For every concept, a separate card (available in two font sizes) with a brief description was presented. Concepts were presented with a minimal use of jargon and illustrated orally with examples. Participants indicated whether they were able (because of low vision) and/or willing to use the cards; seven participants chose to use the concept cards. An overview of the concept cards, including descriptions and examples, is available as Supplementary Table S1 online.

If participants, after the interview, asked us for (professional) psychological support (which happened once), they were referred to a genetic counsellor affiliated with the university hospital in which recruitment occurred. For privacy reasons, we did not follow up on the contact between participants and the genetic counsellor.

### Data analysis

Interviews were audio-recorded, transcribed verbatim, pseudonymised and saved on a password-protected server until completion of the full research project. Software program NVivo12 was used to support data analysis. IPA requires a case-by-case analysis of the transcripts that moves from a descriptive to an interpretative level^22^. Initially, every interview was read multiple times, while descriptive, linguistic and conceptual annotations were made. Secondly, emerging themes were identified inductively. The emerging themes were listed in a table and connections were determined. Every transcript was analysed separately in this way, while always allowing for the appearance of new themes. Lastly, a framework of all themes and subthemes, thematic connections, definitions, and significant excerpts was composed to facilitate an interpretation of the data that exceeded the sum of its parts^22^. As IPA is an interpretative pursuit, an extensive procedure combining peer debriefing and a systematic audit trail was followed to ensure the trustworthiness of both the process and product of analysis^29^. T.M. independently analysed a subset of the data to validate the analysis of M.S. and T.M. and M.S. thoroughly discussed transcripts and theme definitions and connections. Finally, quotes were selected and translated to support the interpretative results.

### Ethics approval

This study is approved by the Commission of Medical Ethics at Ghent University Hospital (reference number B670201628974). The study was conducted in accordance with relevant guidelines and regulations and the principles of the Declaration of Helsinki and subsequent updates. All participants signed an informed consent form which included a statement on the anonymised publication of study results.

## Results

Three themes were identified in the inquiry of patients’ interpretation of UFs: A. Result-specific qualities, B. Lived illness experiences, C. Family embedding.

These themes correspond with the main components of UFs’ meaning from a patient perspective and with underlying motives for (not) wanting to know particular results. Table 1 presents the main findings regarding the components of UFs’ meaning, as well as illustrative quotes.

**Table 1:** Main findings and illustrative quotes regarding the components of UFs’ meaning from a patient perspective.

A. Result-specific qualities		
**A1. Characteristics of the UF**		
Actionability	• Broad interpretation exceeding medical level (*Quote*) • Dynamic and context-dependent	*Then you know that there are some things to keep in mind. […] Like for Alzheimer's disease…, then I could prepare for that and maybe […] pay attention to or notice first symptoms.* (Participant 2)
Penetrance	• Divergent interpretations • Preference for highly penetrant UFs But: difficult to determine cut-off threshold	
Severity	Particular interest in severe diseases But: affected by actionability and burden on others	
Age of onset	Divergent preferences • Young participants: conditions with onset during active life (*Quote*) • Older participants: later-onset conditions ⇒ “As soon as optimally actionable”	*When you're 70 […], I think you're going to have some problems anyway, for instance a cardiac disease. So whether you need to know additional risks then… I think, for me, the limit is about the age of retirement, when life is more relaxed anyway. From that age on, it would matter less to me whether or not I would know [about an UF]. They can tell me but I wouldn’t worry too much. But before you’re 65, you’re still working, you want children or you have young children… Then I would certainly like to know, yeah, for sure.* (Participant 1)
**A2. Consequences of disclosure**		
Operational	• Related to actionability • General willingness to take action But: context-dependent and no guaranteed success (*Quote*)	*It’s all just a matter of definition. […] Some cancers are treatable but what kind of effect do you really realise? 20% of treated patients may live a year longer, so technically, it’s treatable. But actually, in terms of quality of life… Ok, 20% of them get an extra year, but 80% of them don’t.* (Participant 5)
Psychological	Possibility of distress ⇒ On the one hand: preference of partly open future (*Quote*) ⇒ On the other hand: no avoidance of actual risk by not disclosing UFs (*Quote*) But: assumed ability to cope with UFs	*At work, someone died very unexpectedly. But something like that can happen and it can also happen to me: I can get an unexpected disease. However, you have to accept it, you have to move forward* (Participant 11) *If they told me they would only disclose the identified things [UFs] for which a treatment exists, I would be worried and I would wonder what other things they have found but don’t tell.* (Participant 6)

B. Lived illness experiences		
B1. Symptomatic experience		
	• Distress by unpredictable IRD prognosis But: sometimes preferring the unknown (*Quote*) • Ubiquitous impact of IRD	*Q: Suppose there would be a crystal ball about your IRD prognosis, would you want to look into it?* *A: That’s a difficult one… I don't know. If the answer would be that I would suffer severe visual impairment only at a later age, then I'd like to know. But if it would be within ten years… (*Participant 4)
B2. Diagnostic focus		
	• Specific interest in diagnostic genetic test results But: relative importance of genetic diagnosis (*Quote*) • UFs as valuable side-effects (especially when related to IRD) (*Quote*) ⇒ Little interest in SFs	*I don’t get up every day thinking something like “I hope they’re going to find something [a treatment] today”. I know they’re working on it but they were already working on it fifteen years ago. It’s good that they have identified the gene but I realise that scientific research takes a lot of time and money, which are not always available.* (Participant 6) *I have a certain birthmark and it has already been suggested that this may be related with my eye condition. […] So if they take a broader look [in a genetic test], they may find things that are related in some way. I think that’s really a positive thing.* (Participant 6)
B3. Abstract information		
	UFs as both valuable and abstract (*Quote*) ⇒ Symptomatic echoing to overcome abstract, presymptomatic information • Nevertheless: suspicion towards preventive actions concerning UFs (*Quote*)	*My eye condition is really concrete and tangible and I can specifically say “this is my problem, that’s the cause, those are the consequences.” […] But if they’d inform me about a potential risk for breast cancer […], it’s not concrete and present yet. It’s something different than actually having the disease. It’s different to have a predisposition with the chance of not getting the disease.* (Participant 6) *I wouldn't go to the hospital without any problems. If I would feel something, for instance in my breasts, I would ask the doctor for further tests. But for the time being, I have no problems, except for my eyes.* (Participant 11)

C. Family embedding		
C1. Disease prognosis		
	• Family IRD progress as preview, family IRD coping as example (*Quote*) • Family history of (non-IRD associated) disease as stimulus for interest in associated UFs (*Quote*)	*I saw it with my grandfather. Even at his age, he was still able to work with a computer and […] thanks to technology, he could live rather independently. […] He couldn’t see anything but he still enjoyed many things. […] He was really an example to me.* (Participant 4) *For those diseases that are common in my family, I would really like to know [whether I have a predisposition]. I also think there should be a kind of motivation to really take action, like a family member who has cancer or Alzheimer’s disease. Yes, I think there needs to be this kind of incentive first.* (Participant 1)
C2. Family relevance of genetic information		
	Family relevance of IRD-related test results ↔ Limited family relevance of UFs (*Quote*)	*On the one hand, I think it may be good to know for which diseases I have a predisposition. But on the other hand, maybe I prefer not to know all of this and to see whether it ever comes to that. But then again, I think this is a bit contradictory […]. I don’t want my children to have IRD. But for other diseases, I wouldn't do the same. It’s so difficult to choose one side or the other.* (Participant 4)

Most participants oscillated between wanting to know and not wanting to know specific UFs and they balanced various motives, including references to all three components of UFs’ meaning. Moreover, their interpretation often became more nuanced throughout the interview. In what follows, each component of UFs’ meaning from a patient perspective will be analysed in more detail.

### A. Result-specific qualities

The first component refers to potential result-specific qualities of UFs. These qualities include both characteristics of the UF and consequences of disclosure. Important to mention is that none of the participants actually received UFs, hence this component encompassed hypothetical reflections.

#### A1. Characteristics of the UF

Considering UFs and their associated conditions, participants reflected in a nuanced way on characteristics of actionability, penetrance, severity, and age of onset.

Actionability: Firstly, the actionability of an UF’s associated condition was usually interpreted in a broad way that exceeded medical actions. Receiving UFs was considered an opportunity to take clinical but also practical action (concerning, for instance, financial or residential issues), change one’s lifestyle, improve one’s self-awareness or enjoy life to the fullest. Several participants also characterised a condition’s actionability as dynamic and context-dependent, since it may change by scientific progress and living conditions.

Penetrance: Secondly, most participants would prefer to only be informed about “real risks” and hence to only receive highly penetrant UFs with a high probability to result in the associated conditions. Low-penetrance UFs may be numerous and result in (unnecessary) psychological distress or actions. Nevertheless, a cut-off threshold was difficult to determine. Penetrance was variedly interpreted in terms of percentages (“80% chance”), by comparison with other disease risks (“your top 10 of risks”), or by comparison to other people’s perceived risks (“a higher chance than someone else”). An UF’s penetrance was also considered to increase when someone has an associated family history of disease.

Severity: Thirdly, the severity of a condition was an ambivalent motive for disclosure. Overall, patients were particularly interested in preconditions for severe diseases, since these results are most likely to stimulate preventive actions, and some patients claimed that these results should always be reported. Nevertheless, several participants mentioned that the disclosure of UFs regarding less severe conditions could also be useful, as long as it allowed for action in the near future. One participant added that a condition’s severity does not only include personal impact but also its burden on partners or relatives.

Age of onset: Fourthly, patients valued a condition’s age of onset in different ways. Two young participants preferred to receive UFs associated with conditions with an onset during active life (regarding work, reproduction, etc.). These conditions were assumed to have the most substantial impact, whereas later-onset conditions may concur with ”normal ageing”. A second argument against the disclosure of UFs regarding later-onset conditions involved the warning that from the time of disclosure on, this knowledge may be psychologically distressing. Conversely, two participants between 40 and 50 years old expressed their interest in UFs associated with later-onset conditions. Experiences with illness and death in friends or relatives made them more concerned about illness and moreover, these results could be relevant for relatives. The interpretation of a condition’s age of onset was summarised in a participant’s remark that UFs should be reported as soon as they are optimally actionable. Nevertheless, participants doubted the possibility of accurately predicting a condition’s age of onset.

Finally, many participants mentioned the interaction between an UF’s characteristics. An UF-associated condition should, for example, not only be severe but also highly penetrant. Alternatively, low penetrance could be countered by severity or high actionability.

### A2. Consequences of disclosure

Participants were convinced that a disclosed UF has its consequences, either operational (i.e. on actions) or psychological. The specific consequences were assumed to depend on the characteristics of the UF and on someone’s character and context.

Operational consequences: Operational consequences were strongly related to UFs’ actionability and participants showed a general willingness to take medical or personal action. Some patients suggested that if an UF allows for preventive action, especially concerning reproduction, people *should* consider actions which might avoid illness in future children, such as assisted reproduction by in vitro fertilization (followed by embryo selection). On the other hand, actionability was also interpreted context-dependent. In the context of IRD, some patients had experienced that an intention to take action cannot always be realised, for instance because of financial or family reasons. By analogy, they acknowledged that not everyone will take action regarding UFs. Additionally, actions may not always realise the expected outcome. This lack of guaranteed success could motivate not to want to know UFs or to relativize medical actionability.

Psychological consequences: Patients repeatedly mentioned that, in essence, the disclosure of UFs always implies bad news and hence will cause psychological distress. Moreover, disclosure may cause a constant waiting for the first disease symptom, even if disease might never occur. In line with this idea and despite the desire to receive particular UFs, many participants preferred a partly open and unknown future. They considered (health) risks inherent to life and being aware of too many risks may be paralysing and even decrease the actionability of knowledge, since it is impossible to act upon every risk. Therefore, life should also be taken as it comes. On the other hand, patients noticed that refusing UFs only avoids psychological distress but not the actual, physical risk. Therefore, some preferred to know rather than not to know. Despite UFs’ psychological impact, most participants considered themselves able to handle these results. Several participants mentioned specific reasons for this assumed ability, usually related to their (scientific, medical, etc.) job or studies. Remarkably, “other people” were not always supposed to be able to cope with UFs and the importance of genetic counselling for successful coping was repeatedly emphasised.

### B. Lived illness experiences

The second component of UFs’ meaning is based on the difference between, on the one hand, lived experiences of symptomatic illness and diagnostic test results and, on the other hand, reflections on presymptomatic conditions and abstract test results. This component contains three subcomponents: symptomatic experience, diagnostic focus and abstract information. It will be shown that reflections on abstract information regarding presymptomatic UFs are affected by symptomatic illness experiences and a diagnostic focus, and that the interaction between the three subcomponents affects patients’ valuation of preventive actions.

#### B1. Symptomatic experience

All but one participant experienced IRD-associated symptoms: most patients suffered from night blindness and tunnel vision and they had experienced a visual decline. As professionals were not able to provide an exact prognosis, patients considered the evolution of their IRD unpredictable, which was mostly experienced as distressing. Nevertheless, some participants preferred an unknown over a negative prognosis.

The impact of IRD was described as ubiquitous. Participants provided mobility and job-related examples, such as the inability to drive a car or the necessity to change or quit one’s job. Many participants tried to accept the consequences of their illness and emphasised what was still possible. Nevertheless, they regularly met the limits of coping strategies, since many activities were still challenging or impossible.

#### B2. Diagnostic focus

Many patients emphasised that, concerning the IRD-related genetic test, they were specifically interested in diagnostic results. The disclosure of diagnostic test results had generally been appreciated positively, since these results may be a first step towards (future) treatment or be relevant for relatives. Nevertheless, many patients also minimised the importance of diagnostic test results, as these did not change their illness experiences and were merely a confirmation of a clinical diagnosis they already knew. Contrary to their hopes, most patients realised that medical treatment would not be available on short notice.

In line with the diagnostic focus, various patients noted that UFs would be valuable side-effects of a test but not their core interest. Particularly valuable UFs would be those that would be relevant for or related to their IRD. Many patients also would not be interested in diagnostically unrelated UFs if there was no symptomatic reason for a genetic test. Correspondingly, very few participants aspired to actively pursue SFs. As an additional argument, they stated that they were not more at risk for UF- or SF-associated conditions than the general population.

#### B3. Abstract information

Contrary to patients’ diagnostic focus, participants considered the possibility of presymptomatic UFs more ambivalent: these results could be valuable but were also quite abstract.

To overcome the abstract character of presymptomatic UFs, some participants applied a strategy of what we will call “symptomatic echoing”: motives for (not) wanting to know UFs and participants’ interpretation of UFs’ potential qualities echoed elements of IRD experiences. Many patients wanted to receive medically non-actionable UFs and frequently explained this by referring to the medical non-actionability of an IRD and their positive experience with receiving IRD-related test results. Some participants also suggested that their way of managing UFs would be comparable to their IRD-associated coping strategies.

Nevertheless, the difference between diagnostic, IRD-related results and presymptomatic UFs affected participants’ valuation of preventive actions. Many participants had based several (professional, mobility-related, etc.) decisions on assumptions about the evolution of their IRD. Some participants had learned to use a white cane or to read braille, even though they did not need these skills yet. Nevertheless, these preventive actions were considered valuable for possible future situations. Contrarily, the value of preventive actions in function of UFs was questioned sometimes. UFs may never cause actual disease and people suspected the usefulness of reorienting one’s life towards a future that might never happen. Hence, some participants stated that actions concerning UFs could wait until first symptoms would occur.

### C. Family embedding

The last component addresses patients’ embedding in their family context. The impact of family embedding was most clear in patients’ ideas about disease prognosis and about the family relevance of genetic test results.

#### C1. Disease prognosis

When participants had a close family member who was also affected by an IRD, they often perceived this family member’s symptoms and progress as a possible preview of their own illness evolution. Family members with an IRD could partly counter the uncertainty caused by an unknown prognosis and their coping strategies were frequently appraised as valuable examples.

The importance of a family example of illness also appeared in the context of UFs. Many participants indicated a family history of (non-IRD associated) disease, such as cancer or a cardiovascular condition, as a strong stimulus for wanting to know potentially associated UFs. A family example of illness would strengthen the reliability of identified UFs and patients would perceive these findings as not completely unexpected. They also assumed that these findings would motivate to take real action.

#### C2. Family relevance of genetic information

A major concern among participants was the recurrence risk of IRD and therefore, they valued IRD-related test results also because of their potential relevance for (future) children and relatives. Most participants had positive experiences with informing relatives about IRD-related test results. They considered this communication self-evident or even morally required.

Conversely, participants were less concerned about the relevance of potential UFs for relatives and future generations. One participant explicitly acknowledged the difference between his/her strong desire not to pass IRD on to future children and his/her ambivalent interest in UFs.

Most participants indicated that they would only inform their partner and first degree relatives about UFs. Few felt responsible to inform the extended family and they expected this communication to be rather difficult, since UFs have an abstract (because usually presymptomatic) character. Hence, participants feared that family members could deem these results irrelevant or even unwanted.

## Discussion

This study investigated the meaning of UFs in a diagnostic context from the perspective of adult patients with an IRD. This meaning was composed of three components that referred to qualities of the UF itself, patients’ lived illness experiences and family embedding. The components frequently interacted, resulting in a complex meaning structure and nuanced motives regarding disclosure. This nuanced perspective on UFs and SFs has been reported before and is an important correction to the assumption of an unspecified interest in all genomic information^12, 14, 19^.

The first component related to UFs’ result-specific qualities. Patients emphasised the actionability, penetrance, severity and age of onset of the UF-associated condition, characteristics that have been emphasised before by both patients and professionals^6, 13, 15, 21^. However, an accordance between both stakeholders’ ideas should be perceived with caution, since they may interpret these characteristics differently. Most patients interpreted actionability in a broad sense of personal utility that largely exceeded medical interventions. The interest in personally useful results has been expressed in other patient-focused studies but contravenes the disclosure preferences of most professionals^9, 11, 12, 15^. Moreover, it contravenes disclosure preferences of some cancer patients having less interest in medically non-actionable UFs, possibly because of a focus on therapeutic actions^3, 16, 30^. Many participants of our study also expressed ambiguous ideas on UFs’ operational (action-related) consequences. In line with existing literature, they seemed convinced to take preventive action in accordance with genomic information^13^. Nevertheless, the abstract character of UFs may attenuate patients’ motivation^12^. Some participants characterised UFs’ actionability as a quality that would be mainly valuable when the UF actually results in disease. This way, UFs’ presymptomatic character may result in a lower uptake of preventive actions than suggested, an idea that aligns with the moderate operational impact of genetic and genomic information in general^31, 32^. Participants’ remark that there is no guaranteed success of actions may additionally weaken UFs’ operational consequences. As a hypothesis, this remark may be justified by symptomatic echoing and experienced confrontations with the limits of coping strategies for IRD.

Concerning the psychological consequences of UFs, the possibility of distress has been identified as an important motive against disclosure in this and other studies^13, 16, 33^. People may not want to receive too many UFs and, instead, prefer a partly open future^30, 34^. This desire could mirror some patients’ preference of an uncertain over a negative IRD prognosis. On the other hand, our study participants, as well as participants of several cancer-focused studies, generally trusted their own ability to psychologically cope with UFs^18, 20^. This confidence might be caused by the idea of having a better genetic literacy than “the standard patient” and by feelings of resilience and self-efficacy based on previous disease management^12, 18^. The assumption that the disclosure of UFs will be similar to the neutrally or positively experienced disclosure of diagnostic findings may further support participants’ confidence^35, 36^.

The second component of UFs’ meaning refers to patients’ lived illness experiences, an element of which the importance had already been suggested. On the one hand, existing literature claimed that, for instance, cancer and rare disease patients’ focus is on symptomatic conditions and diagnostic test results rather than on UFs or SFs^12, 19, 30, 37^. On the other hand, several studies suggested that a diagnostic focus may also increase (parents of) patients’ interest in UFs and SFs, especially when they did not receive diagnostic test results yet^9, 10, 20, 34^. These people may hope for UFs that could be associated with their symptomatic condition and hence provide a (partial) diagnosis^9, 10, 20^. Our study, however, does not confirm this interest in UFs because of an unsolved diagnostic quest. With one exception, our study included patients who already received a genetic diagnosis. Still, they expressed an interest in UFs and even particularly in UFs that may be relevant for their IRD-diagnosis. Hence, we suggest that patients’ diagnostic focus and the disclosure of a genetic diagnosis should not be interpreted as, respectively, an absolute catalyst or inhibitor of patients’ interest in UFs. Rather, patients’ symptomatic and diagnostic focus contribute, together with specific qualities of the UF and patients’ family embedding, to people’s particular interest in UFs.

Thirdly, patients’ family embedding affected UFs’ interpretation, especially concerning disease prognosis and genetic information’s family relevance. A patient’s family history of disease could provide valuable examples concerning disease prognosis for both IRD and UFs-associated diseases. This way, patients’ family embedding could stimulate their interest in specific UFs, an idea that has been suggested before^10, 12, 19, 21^. This interest may obviously be triggered by a higher risk for illness. Moreover, it could be considered a family-wide variant of symptomatic echoing where family-wide illness experiences counter the abstract character of presymptomatic UFs. This hypothesis is supported by the claim that subjective perceptions of disease risk are driven more by family history than by objective data^38^. Finally, patients made a difference between diagnostic test results’ and UFs’ family relevance and, hence, they considered the impact of family embedding differently in both contexts. Whereas most patients were convinced of the family relevance of diagnostic test results, they were generally less concerned about UFs’ family relevance and they would be less inclined to communicate them to relatives. UFs’ abstract character may partially explain patients’ restricted awareness of UFs’ potential family relevance^12^. Also concerns about bringing bad news to family members have been identified before but these conflict with studies that reported a strong interest in UFs because of their potential value for family and (future) children^20, 30, 37, 39^.

UFs’ complex meaning inherently affects effective counselling procedures. Generally, patients must be granted enough time to carefully consider UFs’ meaning and potential consequences^37^. To avoid an information overload, binning systems, which categorize UFs according to their characteristics and possible impact, are frequently used counselling strategies^40^. However, divergent interpretations of UFs’ qualities challenge the implementation of such binning systems. Professionals and patients particularly differ concerning their understanding of UFs’ actionability since they tend to consider, respectively, medical actionability and personal utility as necessary or sufficient criteria for disclosure^15, 19, 41, 42^. Enabling the disclosure of medically non-actionable findings may, however, raise problems since personal utility may become an unspecified umbrella concept that stimulates the disclosure of any possible UF^43, 44^. To overcome the difficulties concerning medical actionability and personal utility, it has been suggested to discard these characteristics and, instead, consider UFs’ pathogenicity as a sufficient criterion for disclosure^45^.

Moreover, UFs’ complex meaning from a patient perspective implies that counselling should not only address UF-specific qualities but also patients’ illness experiences and family context and, hence, be personalised and context-dependent^30, 42^. For instance, patients’ diagnostic focus suggests that they differentiate a clinical context from a screening opportunity. Counselling procedures should acknowledge this diagnostic focus but should also help patients to overcome the abstract character of UFs and help them understand the potential value of preventive actions for presymptomatic findings.

To our knowledge, this is the first IPA-study on UFs’ meaning from the perspective of adult patients with a monogenic disease. Some limitations should be mentioned however. Participants were selected by genetic professionals/treating physicians. They may have excluded persons who they considered unsuitable for participation because of linguistic, psychological or other reasons, which may have resulted in a biased sample. Even though all participants had experienced illness and/or genetic testing, they had not actually received UFs. Some explicitly identified this lack of experience as a barrier to an adequate interpretation. However, the prospective interpretation of UFs will probably be similar to clinical situations where patients have to decide on disclosure before genetic results are returned. Finally, more women than men participated in this study. No gender differences were observed but the research design of this study does not allow for a meaningful identification of gender-based differences.

## Supplementary Information

Supplementary Information.

## Data Availability

None of the data generated and analysed during this study are publicly available for reasons of personal privacy, but they are available from the corresponding author in response to a reasonable request.

## References

[1] Bertier G, Hetu M, Joly Y (2016). Unsolved challenges of clinical whole-exome sequencing: a systematic literature review of end-users' views. BMC Med. Genomics.

[2] Demougeot L (2018). Changes in clinical practice related to the arrival of next-generation sequencing in the genetic diagnosis of developmental diseases. Arch. Pediatr..

[3] Meiser B, Storey B, Quinn V, Rahman B, Andrews L (2016). Acceptability of, and information needs regarding, next-generation sequencing in people tested for hereditary cancer: a qualitative study. J. Genet. Couns..

[4] Mackley MP, Capps B (2017). Expect the unexpected: screening for secondary findings in clinical genomics research. Br. Med. Bull..

[5] Consugar MB (2015). Panel-based genetic diagnostic testing for inherited eye diseases is highly accurate and reproducible, and more sensitive for variant detection, than exome sequencing. Genet. Med..

[6] van El CG (2013). Whole-genome sequencing in health care Recommendations of the European Society of Human Genetics. Eur. J. Hum. Genet..

[7] Kalia SS (2017). Recommendations for reporting of secondary findings in clinical exome and genome sequencing, 2106 update (ACMG SF v2.0): a policy statement of the American College of Medical Genetics and Genomics. Genet. Med..

[8] Rini C (2018). The who, what, and why of research participants' intentions to request a broad range of secondary findings in a diagnostic genomic sequencing study. Genet. Med..

[9] Bishop CL, Strong KA, Dimmock DP (2017). Choices of incidental findings of individuals undergoing genome wide sequencing, a single center's experience. Clin. Genet..

[10] Shahmirzadi L (2014). Patient decisions for disclosure of secondary findings among the first 200 individuals undergoing clinical diagnostic exome sequencing. Genet. Med..

[11] Kaphingst KA (2016). Preferences for return of incidental findings from genome sequencing among women diagnosed with breast cancer at a young age. Clin. Genet..

[12] Mackley MP (2018). Views of rare disease participants in a UK whole-genome sequencing study towards secondary findings: a qualitative study. Eur. J. Hum. Genet..

[13] Bijlsma RM (2018). Cancer patients' intentions towards receiving unsolicited genetic information obtained using next-generation sequencing. Fam. Cancer.

[14] Yushak ML (2016). Patient preferences regarding incidental genomic findings discovered during tumor profiling. Cancer.

[15] Green RC (2013). ACMG recommendations for reporting of incidental findings in clinical exome and genome sequencing. Genet Med..

[16] Mackley MP, Fletcher B, Parker M, Watkins H, Ormondroyd E (2017). Stakeholder views on secondary findings in whole-genome and whole-exome sequencing: a systematic review of quantitative and qualitative studies. Genet. Med..

[17] Roche MI, Berg JS (2015). Incidental findings with genomic testing: implications for genetic counseling practice. Curr. Genet. Med. Rep..

[18] Hamilton JG (2017). Interest and attitudes of patients with advanced cancer with regard to secondary germline findings from tumor genomic profiling. J. Oncol. Pract..

[19] Boardman F, Hale R (2018). Responsibility, identity, and genomic sequencing: A comparison of published recommendations and patient perspectives on accepting or declining incidental findings. Mol. Genet. Genom. Med..

[20] Hitch K (2014). Lynch syndrome patients' views of and preferences for return of results following whole exome sequencing. J. Genet. Couns..

[21] Bennette CS (2013). Return of incidental findings in genomic medicine: Measuring what patients value-development of an instrument to measure preferences for information from next-generation testing. Genet. Med..

[22] Smith, J. A. & Osborn, M. Interpretative phenomenological analysis. In *Qualitative Psychology: A Practical Guide to Research Methods* (ed Jonathan A Smith) (Sage, 2003).

[23] Osborn M, Smith JA (2006). Living with a body separate from the self. The experience of the body in chronic benign low back pain: an interpretative phenomenological analysis. Scand. J. Caring Sci..

[24] Michie S, Smith JA, Senior V, Marteau TM (2003). Understanding why negative genetic test results sometimes fail to reassure. Am. J. Med. Genet. A.

[25] Cremers FPM, Boon CJE, Bujakowska K, Zeitz C (2018). Special Issue introduction: inherited retinal disease: novel candidate genes, genotype-phenotype correlations, and inheritance models. Genes.

[26] RetNet, https://sph.uth.edu/retnet/disease.htm.

[27] Sahel JA, Dalkara D (2019). Gene therapy for retinal dystrophy. Nat. Med..

[28] Ledford H (2017). FDA advisers back gene therapy for rare form of blindness. Nat. News.

[29] Creswell JW, Miller DL (2000). Determining validity in qualitative inquiry. Theory Practice.

[30] Bijlsma RM (2018). Managing unsolicited findings in genomics: A qualitative interview study with cancer patients. Psycho-Oncol..

[31] Rego S, Dagan-Rosenfeld O, Bivona SA, Snyder MP, Ormond KE (2019). Much ado about nothing: A qualitative study of the experiences of an average-risk population receiving results of exome sequencing. J. Genet. Couns..

[32] Christensen KD, Green RC (2013). How could disclosing incidental information from whole-genome sequencing affect patient behavior?. Pers. Med..

[33] Mighton C (2020). Quality of life drives patients' preferences for secondary findings from genomic sequencing. Eur. J. Hum. Genet..

[34] Houdayer F (2019). Secondary findings from next generation sequencing: Psychological and ethical issues. Family and patient perspectives. Eur. J. Med. Genet..

[35] Vears DF, Dunn KL, Wake SA, Scheffer IE (2015). "It's good to know": experiences of gene identification and result disclosure in familial epilepsies. Epilepsy Res..

[36] Sie AS (2015). Patient experiences with gene panels based on exome sequencing in clinical diagnostics: high acceptance and low distress. Clin. Genet..

[37] Rost C, Dent KM, Botkin J, Rothwell E (2020). Experiences and lessons learned by genetic counselors in returning secondary genetic findings to patients. J. Genet. Couns..

[38] Underhill ML, Lally RM, Kiviniemi MT, Murekeyisoni C, Dickerson SS (2012). Living my family's story: Identifying the lived experience in healthy women at risk for hereditary breast cancer. Cancer Nurs..

[39] Vornanen M (2018). "I would like to discuss it further with an expert": a focus group study of Finnish adults' perspectives on genetic secondary findings. J. Commun. Genet..

[40] Berg JS, Khoury MJ, Evans JP (2011). Deploying whole genome sequencing in clinical practice and public health: Meeting the challenge one bin at a time. Genet. Med..

[41] Isidor B (2019). Searching for secondary findings: considering actionability and preserving the right not to know. Eur. J. Hum. Genet..

[42] Jamal L (2017). When bins blur: Patient perspectives on categories of results from clinical whole genome sequencing. AJOB Empirical Bioethics.

[43] Urban A, Schweda M (2018). Clinical and personal utility of genomic high-throughput technologies: perspectives of medical professionals and affected persons. New Genet. Soc..

[44] Ploug T, Holm S (2017). Clinical genome sequencing and population preferences for information about 'incidental' findings—From medically actionable genes (MAGs) to patient actionable genes (PAGs). PLoS ONE.

[45] Vears D (2018). Points to consider for laboratories reporting results from diagnostic genomic sequencing. Eur. J. Hum. Genet..

