# Supplementary material for: A qualitative study among patients with an inherited retinal disease on the meaning of genomic unsolicited findings
Source: Sci Rep. 2021 Aug 4;11:15834. doi: 10.1038/s41598-021-95258-2 (PMC8339116; doi:10.1038/s41598-021-95258-2)
Supplement: Supplementary file 1 — Supplementary Information. [file 41598_2021_95258_MOESM1_ESM.pdf]

A qualitative study among patients with an inherited retinal disease on the meaning of genomic unsolicited findings

Marlies Saelaert, Heidi Mertes, Tania Moerenhout, Caroline Van Cauwenbergh, Bart P. Leroy, Ignaas Devisch, Elfride De Baere

Theoretical concept	Text mentioned on the card	Examples mentioned orally
**Actionability**	Possibility to potentially prevent the condition (by medical follow-up, by personal behaviour, etc.)	Hereditary breast and ovarian cancer
**Actionability**	Possibility to treat the condition	Arrythmia, such as Long QT syndrome *versus* Alzheimer's disease
**Penetrance**	Chance that the condition will actually develop	Hereditary breast and ovarian cancer *versus* hereditary colon cancer syndromes
**Age of onset**	Estimated age at which the condition might actually develop	Huntington disease or hereditary breast and ovarian cancer *versus* early onset eye condition or Duchenne muscular dystrophy
**Clinical significance**	Exact clinical meaning of the variant/abnormality in a person's DNA/genetic material	Variant of unknown significance in a gene associated with hereditary cancer
**Carrier status**	Potential relevance of the finding not for oneself but for relatives and future generations	Cystic fibrosis
**Frequency**	A frequent or a rare disease	Hereditary breast and ovarian cancer *versus* thyroid cancer
**Severity**	Impact of the condition on someone's (quality of) life	Huntington disease *versus* high blood pressure
**Financial burden**	Financial impact<br>• of the genetic test<br>• of the condition	Hereditary cancer *versus* high blood pressure

Supplementary Table S1: Overview of concept cards used during patient interviews
